# Heat Priming of Lentil (*Lens culinaris* Medik.) Seeds and Foliar Treatment with γ-Aminobutyric Acid (GABA), Confers Protection to Reproductive Function and Yield Traits under High-Temperature Stress Environments

**DOI:** 10.3390/ijms22115825

**Published:** 2021-05-29

**Authors:** Anjali Bhardwaj, Kumari Sita, Akanksha Sehgal, Kalpna Bhandari, Shiv Kumar, P. V. Vara Prasad, Uday Jha, Jitendra Kumar, Kadambot H. M. Siddique, Harsh Nayyar

**Affiliations:** 1 Department of Botany, Panjab University, Chandigarh 160014, India; bhardwajanjali96@gmail.com (A.B.); sitas191@gmail.com (K.S.); as5002@msstate.edu (A.S.); kalpna.bhandari@gmail.com (K.B.); 2 Department of Plant and Soil Sciences, Mississippi State University, Starkville, MS 39762, USA; 3 Biodiversity and Crop Improvement Program, International Center for Agricultural Research in the Dry Areas (ICARDA), Rabat 10112, Morocco; sk.agrawal@cgiar.org; 4 Department of Agronomy, Kansas State University, Manhattan, KS 66506, USA; vara@ksu.edu; 5 Crop Improvement Division, Indian Institute of Pulses Research, Kanpur, Uttar Pradesh 208024, India; u9811981@gmail.com (U.J.); jitendra73@gmail.com (J.K.); 6 The UWA Institute of Agriculture, The University of Western Australia, Perth, WA 6009, Australia; kadambot.siddique@uwa.edu.au

**Keywords:** high temperature, legumes, lentil, pollen, pods, seeds, stress

## Abstract

Gradually increasing temperatures at global and local scales are causing heat stress for cool and summer-season food legumes, such as lentil (*Lens culinaris* Medik.), which is highly susceptible to heat stress, especially during its reproductive stages of development. Hence, suitable strategies are needed to develop heat tolerance in this legume. In the present study, we tested the effectiveness of heat priming (HPr; 6 h at 35 °C) the lentil seeds and a foliar treatment of γ-aminobutyric acid (GABA; 1 mM; applied twice at different times), singly or in combination (HPr+GABA), under heat stress (32/20 °C) in two heat-tolerant (HT; IG2507, IG3263) and two heat-sensitive (HS; IG2821, IG2849) genotypes to mitigate heat stress. The three treatments significantly reduced heat injury to leaves and flowers, particularly when applied in combination, including leaf damage assessed as membrane injury, cellular oxidizing ability, leaf water status, and stomatal conductance. The combined HPr+GABA treatment significantly improved the photosynthetic function, measured as photosynthetic efficiency, chlorophyll concentration, and sucrose synthesis; and significantly reduced the oxidative damage, which was associated with a marked up-regulation in the activities of enzymatic antioxidants. The combined treatment also facilitated the synthesis of osmolytes, such as proline and glycine betaine, by upregulating the expression of their biosynthesizing enzymes (pyrroline-5-carboxylate synthase; betaine aldehyde dehydrogenase) under heat stress. The HPr+GABA treatment caused a considerable enhancement in endogenous levels of GABA in leaves, more so in the two heat-sensitive genotypes. The reproductive function, measured as germination and viability of pollen grains, receptivity of stigma, and viability of ovules, was significantly improved with combined treatment, resulting in enhanced pod number (21–23% in HT and 35–38% in HS genotypes, compared to heat stress alone) and seed yield per plant (22–24% in HT and 37–40% in HS genotypes, in comparison to heat stress alone). The combined treatment (HPr+GABA) was more effective and pronounced in heat-sensitive than heat-tolerant genotypes for all the traits tested. This study offers a potential solution for tackling and protecting heat stress injury in lentil plants.

## 1. Introduction

Air temperatures are rising at both global and local scale, causing heat stress in many cool and summer season food crops and reducing their production potential [1,2]. Heat stress inhibits the growth and development of various food crops by altering several physiological and biochemical processes, which impairs crop performance [2,3]. At the vegetative stage, heat stress causes leaf chlorosis, necrosis, and accelerated phenology, and damages leaf tissue due to membrane injury, denatured proteins, oxidative damage, and dehydration [3]. At the reproductive stage, heat stress severely impacts the plants by disrupting pollen and stigma function and resulting in the lower seed set percentage [2,4]. The process of fertilization is impacted because of obstruction in pollen development, germination, and tube growth, resulting in pod set failure in different food grain and legumes crops [2,5,6].

Lentil (*Lens culinaris* Medik.) is grown as a winter-season food legume and is highly susceptible to heat stress [7]. Lentil requires lower temperatures during vegetative growth and warmer temperatures lead up to maturity; 18–30 °C is considered as its optimum temperature range [8,9]. Lentil is cultivated on large areas in comparatively warmer parts of central and southern India, where supra-optimal temperatures, especially at the time of reproductive stage, significantly inhibit its yield potential. Moreover, global climate changes have shortened the cold period and lengthened the heat periods, further exposing winter-season crops to heat stress. In 2009, a heat wave (35 °C for six days) in south-eastern Australia decreased lentil yields by 70% [10]. In Australia, temperatures more than 32/20 °C (max/min) in course of flowering and pod filling markedly reduced lentil seed yield and quality [10]. Hence, strategies are needed to impart heat tolerance in lentil.

Heat priming refers to a pre-treatment to plants at moderate temperature, which imparts tolerance to subsequent high temperature exposure in plants [11]. Heat priming can also be done to hydrating or germinating seeds. Some studies have indicated the potential benefits of heat priming during the early vegetative stage on plant performance during subsequent high temperature events, as in wheat (*Triticum aestivum* L.) [12], *Arabidopsis* [13], while another study reported no effect of heat priming on wheat performance [11]. Heat tolerance can also be improved with the application of growth-regulating molecules, such as salicylic acid, nitric oxide, polyamines, and others [14]. One of the promising molecules, γ-aminobutyric acid (GABA), a non-protein amino acid, has been shown to act as a signaling molecule in plants [15]. The involvement of GABA in plants exposed to various abiotic and biotic stresses has been reviewed recently [16], and the beneficial effects of GABA have been reported in the past [17,18,19]. Endogenous GABA levels changed in response to stress and influenced the defense mechanisms, pathways and processes [15]. GABA has been implicated in plant cell functioning, such as signaling, osmoregulation, cytosolic pH regulation, buffering in C and N metabolism, and oxidative stress protection [20,21]. Heat stress increased GABA levels and calcium-induced activation of glutamate decarboxylase [22]. GABA is involved in the sexual reproduction of angiosperms and is an essential amino acid for pollen fertility, and plays a vital role in the post-pollination fertilization process [23].

In the present study, we attempted heat-priming the hydrated lentil seeds along with a foliar GABA treatment to assess their effects on reducing the heat injury. Though, earlier studies have reported heat priming the plants at vegetative stages as an approach to induce subsequent stress tolerance, we tried this method on hydrated seeds in a controlled environment, to test its feasibility and to develop it as a technique to counter the effects of high temperature at later growth stages of lentil plants. The objective was to test the effectiveness of heat priming (HPr; 6 h at 35 °C) the lentil seeds and a foliar treatment of γ-aminobutyric acid (GABA; 1 mM; applied twice at different times), singly or in combination (HPr+GABA), under heat stress (32/20 °C) in two heat-tolerant (HT; IG2507, IG3263) and two heat-sensitive (HS; IG2821, IG2849) genotypes to mitigate heat stress. It was hypothesized that a short heat treatment of the hydrated seeds, when applied in combination with foliar GABA treatment, might enhance the heat tolerance in lentil. We probed their effects on several traits related to growth and yield, as well as defense mechanisms involving leaves and flowers.

## 2. Results

### 2.1. Phenology

**Figure 1:**
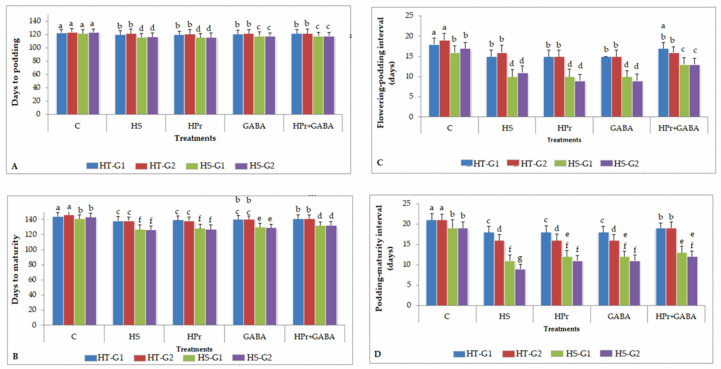
Phenology (Days to podding: (**A**); days to maturity: (**B**); flowering-podding interval: (**C**); podding—maturity interval: (**D**) of heat-tolerant (G1: IG2507; G2: IG3263) and heat-sensitive (G1: IG2821; G2: IG2849) genotypes in control (28/18 °C; 12 h each), heat-stressed (32/20 °C; 12 h each), heat-primed (HPr), GABA-treated, and HPr+GABA treatments. Plants were exposed to heat stress at the onset of flowering (bud stage) for all genotypes; hence, the phenological data related to days to flowering is similar for all treatments. Vertical bars represent standard errors (*n* = 3). Different small letters on the bars indicate significant differences from each other (*p* < 0.05). LSD (least significant difference) for interaction (genotypes × stages × treatments) (*p* < 0.05): Days to podding: 1.8; days to maturity: 1.9; flowering-podding interval: 1.8; podding—maturity interval: 1.7.

Heat stress reduced the days to podding and maturity, more so in heat-sensitive (HS) than heat-tolerant (HT) genotypes (Figure 1A,B). As a result, the flowering to podding and podding to maturity intervals (Figure 1C,D) also decreased significantly, compared to the controls. Heat priming (HPr) alone did not significant affect the phenology. The GABA treatment slightly increased days to flowering and podding and the intervals between different stages. The combined HPr+GABA treatment was more effective at restoring phenology than their individual treatments. The heat-sensitive genotypes responded more to the combined treatment than the heat-tolerant genotypes, with significant improvements in the flowering–podding and podding–maturity intervals.

### 2.2. Stress Injury to Leaves

#### 2.2.1. Membrane Damage

**Figure 2:**
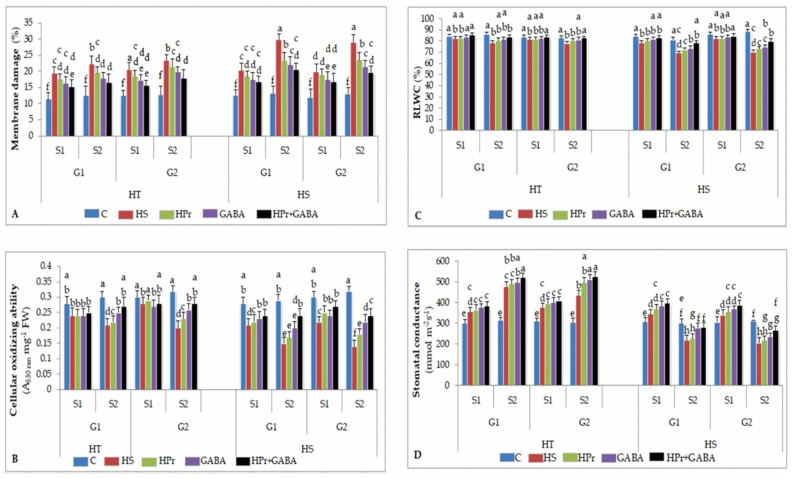
Effect of heat priming (HPr) and γ-amino butyric acid (GABA), applied individually or in combination (HPr+GABA) on membrane damage (**A**), cellular oxidizing ability (**B**), relative leaf water content, RLWC (**C**) and stomatal conductance (**D**) on heat-tolerant (G1: IG2507; G2: IG3263) and heat-sensitive (G1: IG2821; G2: IG2849) genotypes at stage 1 (S1) and stage 2 (S2) in heat-stressed (HS) lentil plants, compared to control (**C**). Vertical bars represent standard errors (*n* = 3). Different small letters on the bars indicate significant differences from each other (*p* < 0.05). LSD for interaction (genotypes × stages × treatments) (*p* < 0.05): membrane damage: 2.9; cellular oxidizing ability: 0.030, relative leaf water content: 2.2, stomatal conductance: 26.3. FW = fresh weight.

The membrane damage as electrolyte leakage (EL) measurements (expressed as percentage) indicated that heat stress caused 19–20% membrane damage during stage 1 and 20–23% damage during stage 2 in HT genotypes, with the corresponding values for HS genotypes being 19–22% and 28–29% (Figure 2A). The HPr and GABA treatments, applied alone, significantly reduced the membrane damage caused by heat stress, compared to control plants. The HPr+GABA treatment further reduced the membrane damage to about 15% during stage 1 in HT genotypes and 17% in HS genotypes, and 16–18% during stage 2 in HT genotypes and 19–20% in HS genotypes.

#### 2.2.2. Cellular Oxidizing Ability

Heat stress reduced cellular oxidizing ability by 6–16% in HT and 25–26% in HS genotypes during stage 1 and 30–38% in HT and 48–56% in HS genotypes during stage 2, compared to the respective controls (Figure 2B). Compared to heat stress, applied singly, HPr significantly increased the oxidizing ability during stage 2 in HS genotypes, while HT genotypes were less responsive at both stages. The GABA treatment increased cellular oxidizing ability by 19–30% during stage 2 in HT genotypes and 33–57% in HS genotypes, compared to heat stress applied singly. The HPr+GABA treatment further increased this trait, by 28–40% in HT and 60–71% in HS genotypes during stage 2, in comparison to heat stress applied singly.

#### 2.2.3. Leaf Water Status

Heat stress decreased leaf water status, recorded as relative leaf water content (RLWC; expressed as percentage), to 81.6–82.5% in HT and 78.4–82.3% in HS genotypes during stage 1, and 78.1–78.5% in HT and 69.4–70.4% in HS genotypes during stage 2, with reference to the controls (83.4–84.5% in HT and 84.3–86.3% in HS genotypes during stage 1, 82.9–86.5% in HT and 81.4–88.5% in HS genotypes during stage 2) (Figure 2C). The HPr and GABA treatments alone significantly improved RLWC, especially in HS genotypes. The HPr+GABA treatment further improved RLWC to 83.4–85.3% in HT and 82.8–84.5 in HS genotypes during stage 1, and to 83% in HT and 78–80% in HS genotypes during stage 2.

#### 2.2.4. Stomatal Conductance

Stomatal conductance (g_s_) increased with heat stress by 18–20% in HT and 11–12% in HS genotypes during stage 1, compared to control plants (Figure 2D). During stage 2, heat stress increased g_s_ by 42–51% in HT genotypes, but decreased it by 27–34% in HS genotypes, in comparison to the controls. The HPr treatment slightly increased g_s_ in both HS and HT genotypes, with reference to heat stress applied singly. The GABA treatment significantly increased g_s_, more so during stage 2, especially in HS genotypes, compared to heat stress alone. In the HPr+GABA treatment, g_s_ increased more in HS (by 14–15% and 28–29%) than HT genotypes (by 8–9% and 10–21%) during stage 1 and stage 2, respectively, with reference to heat stress applied singly.

### 2.3. Reproductive Function

#### 2.3.1. Pollen Germination

**Figure 3:**
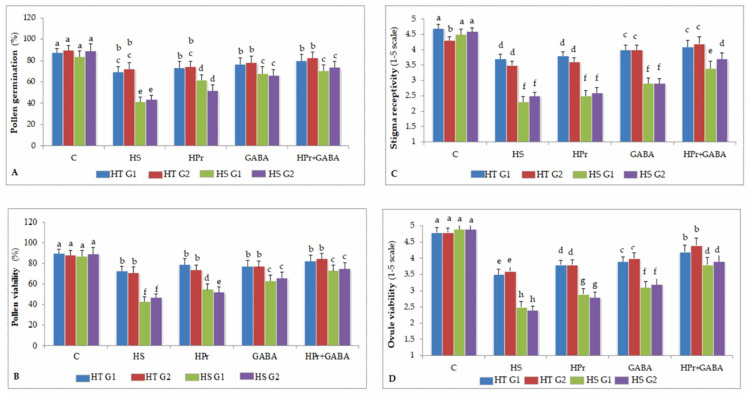
Effect of heat priming (HPr) and γ-amino butyric acid (GABA), applied individually or in combination (HPr+GABA) on pollen germination (**A**), pollen viability (**B**), stigma receptivity (**C**) and ovule viability (**D**) on heat-tolerant (G1: IG2507; G2: IG3263) and heat-sensitive (G1: IG2821; G2: IG2849) genotypes at stage 1 (S1) and stage 2 (S2) in heat-stressed (HS) lentil plants, compared to control (**C**). Vertical bars represent standard errors (*n* = 3). Different small letters on the bars indicate significant differences from each other (*p* < 0.05). LSD for interaction (genotypes × stages × treatments) (*p* < 0.05): pollen germination: 6.2, pollen viability: 6.4, stigma receptivity: 0.25, ovule viability: 0.26.

Compared to pollen germination (PG; expressed as percentage) in control plants (87–89% in HT and 83–88% in HS genotypes), heat stress reduced PG in HS genotypes (41–43%) and HT genotypes (73–74%) (Figure 3A). The HPr treatment improved PG, more so in HS (51–61%) than HT genotypes (73–74%). Similarly, the GABA treatment increased PG more in HS genotypes (65–67%) than HT (76–78%) genotypes. The HPr+GABA treatment increased PG to 70–73% in HS genotypes and 79–82% in HT genotypes.

#### 2.3.2. Pollen Viability

Pollen viability (PV; expressed as percentage) in the control plants was 88–89% in HT and 86–89% in HS genotypes (Figure 3B). Heat stress reduced PV to 70–72% in HT and 42–46% in HS genotypes. The HPr treatment significantly improved PV to 51–54% in HS genotypes, compared to heat stress alone. Treatment with GABA also increased PV in HS genotypes to 62–66%, with reference to heat stress applied singly. The HPr+GABA treatment markedly improved PV to 81–83% in HT and 72–74% in HS genotypes.

#### 2.3.3. Stigma Receptivity

Heat stress decreased stigma receptivity (SR) more in HS genotypes (45–49%) than HT genotypes (19–21%), compared to the controls (Figure 3C). The GABA treatment alone was more effective than the HPr treatment at increasing SR, by 12–26% in HS and 8–14% in HT genotypes, compared to heat treatment alone. The HPr+GABA treatment increased SR by 47–48% in HS genotypes and 11–20% in HT genotypes, compared with heat stress applied singly.

#### 2.3.4. Ovule Viability

Heat stress reduced ovule viability (OV) by 25–27% in HT and 49–51% in HS genotypes, in comparison to the controls (Figure 3D). The HPr treatment increased OV by 8% in HT and 16% in HS genotypes, and the GABA treatment increased OV by 11% in HT and 33% in HS genotypes, compared to the heat treatment alone. The HPr+GABA treatment improved OV by 20–22% in HT and 52–62% in HS genotypes, compared to heat stress alone.

### 2.4. Photosynthetic Function

#### 2.4.1. Photosynthetic Efficiency

**Figure 4:**
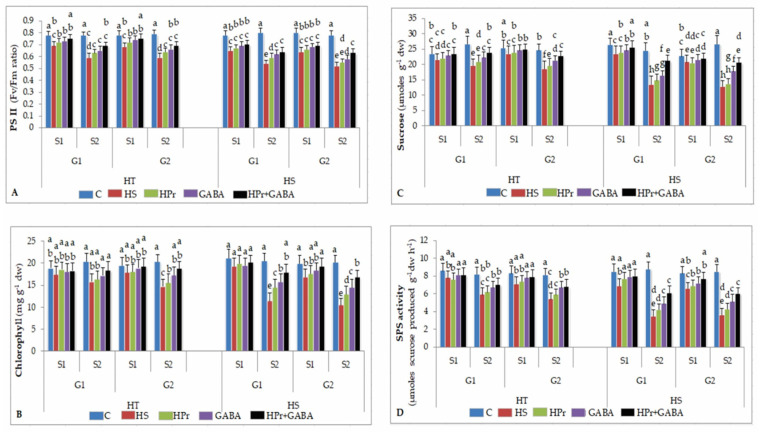
Effect of heat priming (HPr) and γ-amino butyric acid (GABA), applied individually or in combination (HPr+GABA) on photosystem (PS) II function (**A**), chlorophyll content (**B**), sucrose (**C**) and sucrose phosphate synthase, SPS (**D**) on heat-tolerant (G1: IG2507; G2: IG3263) and heat-sensitive (G1: IG2821; G2: IG2849) genotypes at stage 1 (S1) and stage 2 (S2) in heat-stressed (HS) lentil plants, compared to control (**C**). Vertical bars represent standard errors (*n* = 3). Different small letters on the bars indicate significant differences from each other (*p* < 0.05). LSD for interaction (genotypes × stages × treatments) (*p* < 0.05): photosystem II: 0.039, chlorophyll content: 1.9; sucrose: 2.3, sucrose phosphate synthase: 0.91. dw = dry weight.

Photosynthetic efficiency was assessed as chlorophyll fluorescence (ChlF), an indicator of Photosystem II function in the electron transport chain of photosynthesis (Figure 4A). Heat stress, relative to controls, reduced ChlF by 11–13% during stage 1 and 24–25% during stage 2 in the HT genotypes and 17–20% during stage 1 and 32–33% during stage 2 in the HS genotypes, with respect to the controls. The HPr treatment slightly improved ChlF in the HT and HS genotypes, with reference to heat stress applied singly. The GABA treatment significantly increased ChlF, more so in HS than HT genotypes, compared to heat stress alone. The HPr+GABA treatment increased ChlF by 9–10% in HT and 8% in HS genotypes during stage 1, and 17% in HT and 19–21% in HS genotypes during stage 2, in reference to heat stress applied singly.

#### 2.4.2. Chlorophyll

Heat stress had little effect on chlorophyll (Chl) concentration in HT genotypes during stage 1, but significantly decreased it in HS genotypes by 9–15%, compared to the control (Figure 4B). During stage 2, heat stress decreased Chl by 44–48% in HS genotypes and 23–27% in HT genotypes, with respect to the control. The positive effect of HPr and GABA on improving Chl was more noticeable at stage 2. The HPr treatment increased Chl by 24–27% in HS genotypes, and the GABA treatment increased Chl by 37–39% in HS and 9–17% in HT genotypes, compared to heat stress alone. The HPr+GABA treatment caused more increase, which was 57–61% in HS and 17–18% in HT genotypes, with reference to heat stress alone.

#### 2.4.3. Sucrose

Heat stress decreased sucrose (Suc) concentration by 8–9% in HT and 9–11% in HS genotypes during stage 1, and by 24–26% in HT and 45–52% in HS genotypes during stage 2, in comparison to the controls (Figure 4C). The HPr treatment slightly improved Suc in HT and HS genotypes, more so in HS genotypes, compared to heat stress applied alone. The GABA treatment increased Suc by 14–15% in HT and 33–39% in HS genotypes, with reference to heat stress applied singly. The HPr+GABA treatment resulted in more increase in Suc; 37–57% in HS and 21–23% in HT genotypes during stage 2, compared to heat stress alone.

#### 2.4.4. Sucrose Phosphate Synthase

Heat stress reduced SPS activity by 9–14% in HT and 18–20% in HS genotypes during stage 1 and 28–33% in HT and 59–60% in HS genotypes during stage 2, with reference to the control (Figure 4D). The HPr treatment increased SPS activity in HS genotypes by 11.5–17% during stage 1 and 19–20% during stage 2, compared to heat stress applied singly, but had a smaller effect in HT genotypes. The GABA treatment increased SPS activity in HS genotypes by 9–15% during stage 1 and 40–41% during stage 2, with respect to heat stress alone. For HT genotypes, the GABA treatment had no significant effect on SPS activity during stage 1, but increased it by 13–24% during stage 2, in comparison to heat stress applied singly. The HPr+GABA treatment increased SPS activity by 4–11% in HT and 16–17% in HS genotypes during stage 1 and 18–26% in HT and 66–74% in HS genotypes during stage 2, compared to heat stress alone.

### 2.5. Oxidative Damage

#### 2.5.1. Malondialdehyde

**Figure 5:**
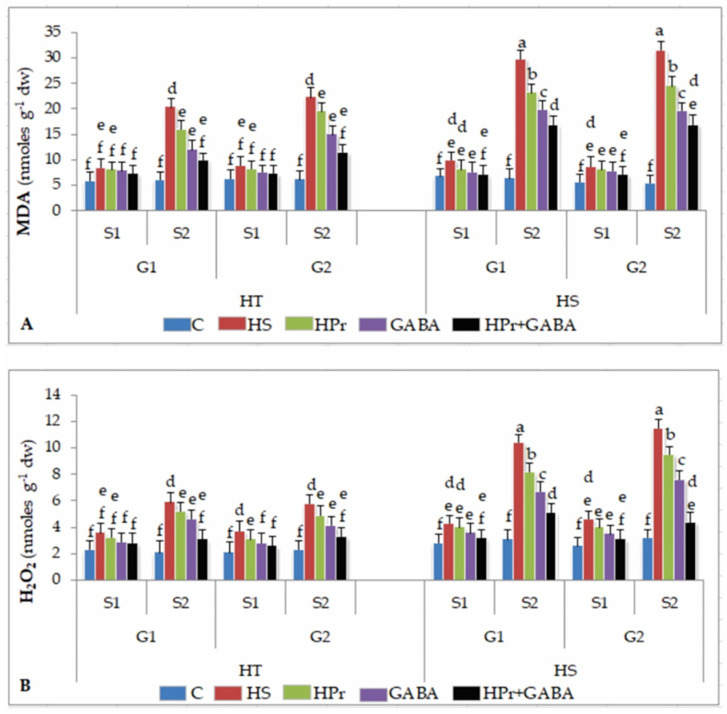
Effect of heat priming (HPr) and γ-amino butyric acid (GABA), applied individually or in combination (HPr+GABA) on malondialdehyde, MDA (**A**) and hydrogen peroxide, H_2_O_2_ (**B**) on heat-tolerant (G1: IG2507; G2: IG3263) and heat-sensitive (G1: IG2821; G2: IG2849) genotypes at stage 1 (S1) and stage 2 (S2) in heat-stressed (HS) lentil plants, compared to control (**C**). Vertical bars represent standard errors (*n* = 3). Different small letters on the bars indicate significant differences from each other (*p* < 0.05). LSD for interaction (genotypes × stages × treatments) (*p* < 0.05): malondialdehyde: 1.9, hydrogen peroxide: 0.74. dw = dry weight.

Lipid peroxidation was measured as malondialdehyde (MDA) concentration. Heat stress increased MDA concentrations by 41–42% in HT and 45–52% in HS genotypes during stage 1, with further increases during stage 2, more so in HS genotypes, in comparison to the controls (Figure 5A). The HPr treatment decreased MDA concentrations by 13–20% in HT and 22% in HS genotypes during stage 2, with respect to heat stress applied singly. The GABA treatment reduced MDA concentrations by 32–40% in HT and 33–37% in HS genotypes during stage 2, compared to heat stress applied singly. On the other hand, the HPr+GABA treatment reduced MDA concentrations by 13–18% in HT and 18–28% in HS genotypes during stage 1 and 40–43% in HT and 51–56% in HS genotypes during stage 2, with reference to heat stress alone.

#### 2.5.2. Hydrogen Peroxide

Heat stress increased H_2_O_2_ concentrations by 56–76% in HT and 53–76% in HS genotypes during stage 1, which increased further during stage 2, more so in HS genotypes, compared to the controls (Figure 5B). The HPr treatment decreased H_2_O_2_ concentrations by 11–16% in HT and 7–13% in HS genotypes during stage 1 and 12–15% in HT and 17–21% in HS genotypes during stage 2, in comparison to heat stress applied singly. The GABA treatment reduced H_2_O_2_ concentrations by 22–29% in HT and 34–35% in HS genotypes during stage 2, compared to heat stress alone. The combined treatment (HPr+GABA) was more effective in reducing H_2_O_2_ concentrations by 43–47% in HT and 51–61% in HS genotypes during stage 2, in comparison to heat stress applied singly.

### 2.6. Antioxidants

#### 2.6.1. Superoxide Dismutase (SOD)

**Figure 6:**
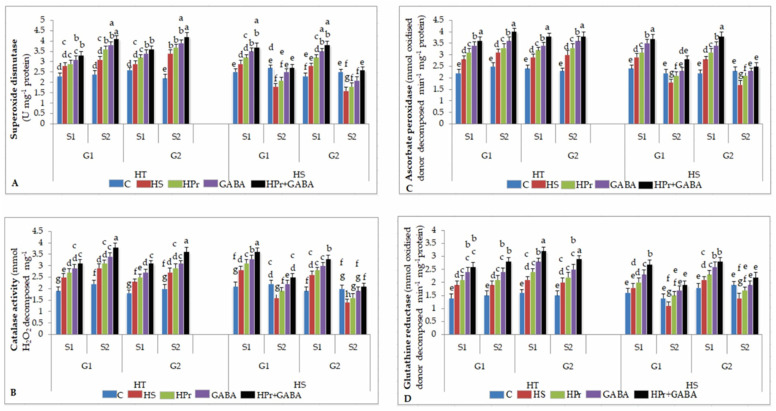
Effect of heat priming (HPr) and γ-amino butyric acid (GABA), applied individually or in combination (HPr+GABA) on superoxide dismutase (**A**), catalase (**B**), ascorbate peroxidase (**C**) and glutathione reductase (**D**) activities on heat-tolerant (G1: IG2507; G2: IG3263) and heat-sensitive (G1: IG2821; G2: IG2849) genotypes at stage 1 (S1) and stage 2 (S2) in heat-stressed (HS) lentil plants, compared to control (**C**). Vertical bars represent standard errors (*n* = 3). Different small letters on the bars indicate significant differences from each other (*p* < 0.05). LSD for interaction (genotypes × stages × treatments) (*p* < 0.05): superoxide dismutase: 0.21, catalase: 0.19; ascorbate peroxidase: 0.20, glutathione reductase: 0.19.

Heat stress increased SOD activity by 12–22% in HT and 16–22% in HS genotypes during stage 1, with reference to the controls; during stage 2, SOD activity increased by 29–54% in HT, but decreased by 33–36% in HS genotypes, compared to the controls (Figure 6A). The HPr and GABA treatments alone increased SOD activity slightly at both stages, more so with the GABA treatment, with respect to heat stress applied singly. Whereas the HPr+GABA treatment increased SOD activity by 18–24% in HT and 27–35% in HS genotypes during stage 1 and 23–32% in HT and 50–62% in HS genotypes during stage 2, in comparison to heat stress alone.

#### 2.6.2. Catalase (CAT)

Heat stress increased CAT activity by 27–31% in HT and 33–37% in HS genotypes during stage 1, compared to the controls; during stage 2, CAT activity increased by 32–35% in HT, but decreased by 30–37% in HS genotypes, compared to the controls (Figure 6B). The HPr and GABA treatments alone significantly increased CAT activity in HS genotypes. The HPr+GABA treatment increased CAT activity by 24–34% in HT and 26–27% in HS genotypes during stage 1 and 31–33% in HT and 50–56% in HS genotypes during stage 2, in comparison to heat stress applied singly.

#### 2.6.3. Ascorbate Peroxidase (APX)

APX activity increased as a result of heat stress, by 21–27% and 24–30% in HT genotypes during stage 1 and stage 2, respectively, compared to the controls; for HS genotypes, APX activity increased by 21–27% during stage 1, but decreased by 18–26% during stage 2, in relation to the controls (Figure 6C). The HPr treatment increased APX activity by about 10% in HT and 16–23% in HS genotypes, with reference to heat stress alone. With GABA treatment, APX activity increased by 17–21% in HT and 20–21% in HS genotypes during stage 1 and 14% in HT and 27–35% in HS genotypes during stage 2, compared to heat stress applied singly. On the other hand, the HPr+GABA treatment increased APX activity by 21–24% in HT and 47–55% in HS genotypes during stage 2, with reference to heat stress alone.

#### 2.6.4. Glutathione Reductase (GR)

Heat stress increased GR activity by 33–35% in HT and 12–16% in HS genotypes during stage 1, and 26–33% in HT and 21–26% during stage 2, with reference to the controls (Figure 6D). The HPr treatment increased GR activity more in HS than HT genotypes, especially during stage 2, which increased by 21–36%, compared to heat stress applied singly. The GABA treatment increased GR activity by 25–26% in HT and 35–54% in HS genotypes during stage 2, with respect to heat stress alone. The HPr+GABA treatment increased GR activity more in HS (57–72% increase) than HT genotypes (45–47%), compared to heat stress applied singly.

### 2.7. Osmolytes

#### 2.7.1. Proline (Pro)

**Figure 7:**
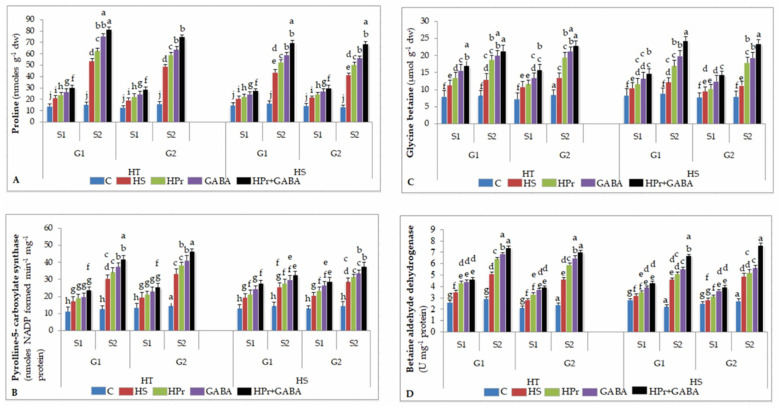
Effect of heat priming (HPr) and γ-amino butyric acid (GABA), applied individually or in combination (HPr+GABA) on proline (**A**) and pyrolline-5- carboxylate synthase (**B**) glycine betaine (**C**) and betaine aldehyde dehydrogenase (**D**) on heat-tolerant (G1: IG2507; G2: IG3263) and heat-sensitive (G1: IG2821; G2: IG2849) genotypes at stage 1 (S1) and stage 2 (S2) in heat-stressed (HS) lentil plants, compared to control (**C**). Vertical bars represent standard errors (*n* = 3). Different small letters on the bars indicate significant differences from each other (*p* < 0.05). LSD for interaction (genotypes × stages × treatments) (*p* < 0.05): proline: 2.7, pyrolline-5-carboxylate synthase: 2.8, glycine betaine: 2.8, betaine aldehyde dehydrogenase: 0.19. dw = dry weight.

Heat stress increased Pro accumulation by 50–56% in HT and 42–50% in HS genotypes during stage 1, in relation to the controls, with further increases during stage 2, more so in HT genotypes (Figure 7A). The HPr treatment increased Pro content by 11–16% in HT and 9–14% in HS genotypes during stage 1, and 17–22% in HT and 21–23% in HS genotypes during stage 2, with reference to heat stress alone. Pro concentration with GABA treatment increased by 25–27% in HT and 19–25% in HS genotypes during stage 1 and 30–32% in HT and 32–35% in HS genotypes during stage 2, in comparison to heat stress applied singly. The HPr+GABA treatment further increased Pro accumulation by 42–47% in HT and 34–40% in HS genotypes during stage 1 and 51–52% in HT and 62–65% in HS genotypes during stage 2, compared to heat stress alone.

#### 2.7.2. Pyrroline-5-Carboxylate Synthase (P5CS)

Heat stress increased P5CS activity by 44–54% in HT and 46–58% in HS genotypes during stage 1 and 133–143% in HT and 77–96% in HS genotypes during stage 2, with reference to the controls (Figure 7B), which contributed to increase in Pro accumulation. The HPr treatment increased P5CS activity by 20–22% in HT and 14–17% in HS genotypes during stage 2, compared to heat stress applied singly. P5CS activity with GABA treatment increased by 33–35% in HT and 23–26% in HS genotypes during stage 2, in comparison to heat stress alone. The combination (HPr+GABA) treatment increased P5CS activity by 30–36% in HT and 29–33% in HS during stage 1 and 39–43% in HT and 38–40% in HS genotypes during stage 2, with reference to heat stress applied singly.

#### 2.7.3. Glycine Betaine (GB)

Heat stress increased GB accumulation by 43–48% in HT and 24–26% in HS genotypes during stage 1 and 52–60% in HT and 36–43% in HS genotypes during stage 2, with respect to the controls (Figure 7C). GB accumulation with HPr treatment increased by 44–46% in HT and 16–29% in HS genotypes during stage 2, with reference to heat stress alone. On the other hand, the GABA treatment increased GB accumulation by 54–57% in HT and 37–40% in HS genotypes during stage 2, compared to heat stress applied singly. The combined treatment (HPr+GABA) increased GB accumulation by 64–69% in HT and 74–76% in HS genotypes during stage 2, with respect to heat stress applied singly.

#### 2.7.4. Betaine Aldehyde Dehydrogenase (BADH)

Heat stress increased BADH activity by 33–34% in HT and 21–24% in HS genotypes during stage 1 and 75–91% in HT and 46–50% in HS genotypes during stage 2, compared to the controls (Figure 7D). With HPr treatment, the BADH activity increased by 17–18% in HT and 11–13% in HS genotypes during stage 1 and 27–28% in HT and 23–26% in HS genotypes during stage 2, in comparison to heat stress alone. The GABA treatment resulted in an increase in BADH activity by 25–32% in HT and 11–13% in HS genotypes during stage 1 and 34–42% in HT and 23–26% in HS genotypes during stage 2, compared to heat stress applied singly. The combination of HPr+GABA treatment increased BADH activity by 31–37% in HT and 28–33% in HS genotypes during stage 1 and 45–53% in HT and 47–56% in HS genotypes during stage 2, in comparison to heat stress alone.

#### 2.7.5. Endogenous GABA

**Figure 8:**
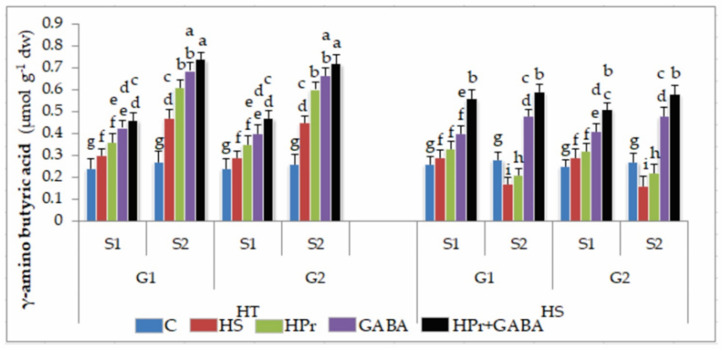
Effect of heat priming (HPr) and γ-amino butyric acid (GABA), applied individually or in combination (HPr+GABA) on endogenous GABA in heat-tolerant (G1: IG2507; G2: IG3263) and heat-sensitive (G1: IG2821; G2: IG2849) genotypes at stage 1 (S1) and stage 2 (S2) in heat-stressed (HS) lentil plants, compared to control (C). Vertical bars represent standard errors (*n* = 3). Different small letters on the bars indicate significant differences from each other (*p* < 0.05). LSD for interaction (genotypes × stages × treatments) (*p* < 0.05): 0.038. dw = dry weight.

Heat stress increased endogenous GABA concentrations by 21–25% in HT and 13–16% in HS genotypes during stage 1 whereas at stage 2, HT genotype showed 73–77% increase in GABA, while a marked reduction (38–40%) was observed in HS genotype at this stage with respect to the controls (Figure 8). The HPr treatment increased endogenous GABA concentrations by 23 and 30–34% at stage 1 and 2, respectively, in HT genotypes while in HS genotypes, endogenous GABA concentration showed 12–14% increase at stage 1 and 48–51% increase at stage 2, compared to heat stress applied singly. The exogenous GABA treatment increased the endogenous GABA concentrations, more so in HS genotypes than HT genotypes during both the stages, referring to heat stress alone. The HPr+GABA combination resulted in additional increase in endogenous GABA concentrations in both the genotypes, to a greater extent in HS genotypes, with reference to heat stress alone.

### 2.8. Yield Traits

#### 2.8.1. Pod Number

**Figure 9:**
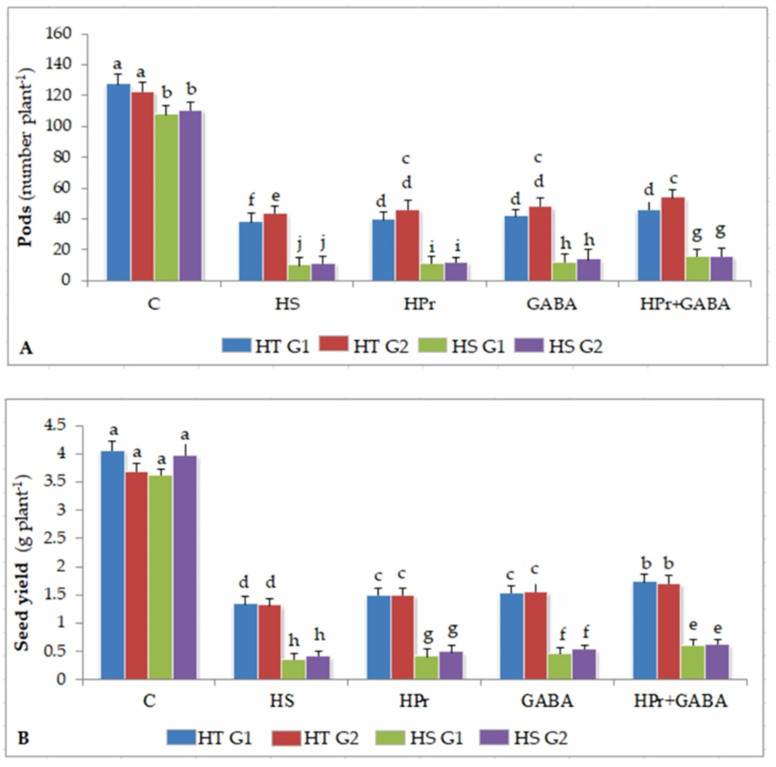
Effect of heat priming (HPr) and γ-amino butyric acid (GABA), applied individually or in combination (HPr+GABA) on pod number (**A**) and seed yield/plant (**B**) in heat-tolerant (HT; G1: IG2507; G2: IG3263) and heat-sensitive (HS; G1: IG2821; G2: IG2849) genotypes in heat-stressed (HS) lentil plants, compared to control (**C**). Vertical bars represent standard errors (*n* = 3). Different small letters on the bars indicate significant differences from each other (*p* < 0.05). LSD for interaction (genotypes × stages × treatments) (*p* < 0.05): pod number: 6.8, seed yield/plant: 0.29.

Heat stress reduced pod number (per plant) more in HS (90%) than HT (64–70%) genotypes, compared to their respective controls (Figure 9A). The HPr treatment had little effect on pod numbers in HT genotypes, but increased it by 13–16% in HS genotypes, compared to heat stress applied singly. The GABA treatment increased pod numbers by about 10% in HT and 21–24% in HS genotypes, in comparison to heat stress alone. The combination of HPr+GABA treatment was more effective and increased pod numbers by 21–24% in HT and 35–38% in HS genotypes, with respect to heat stress applied singly.

#### 2.8.2. Seed Yield

Heat stress decreased seed yield (per plant) by 63–66% in HT and 90–93% in HS genotypes, compared to their respective controls. The HPr treatment increased seed yield by 10–13% in HT and 14–17% in HS genotypes, with respect to heat stress alone (Figure 9B). The GABA treatment increased seed yield by 15–18% in HT and 24–27% in HS genotypes, compared to heat stress applied singly. The combined HPr+GABA treatment increased seed yield by 22–24% in HT and 37–40% in HS genotypes, in comparison to heat stress alone.

## 3. Discussion

The results of this research showed an opportunity to minimize the impact of heat stress on lentil by heat priming the hydrated seeds (to evoke early defense response through the concept of stress memory), followed by application of GABA. The combination of two treatments significantly enhanced the performance of the heat-stressed lentil plants in terms of reduction in stress injury to leaves (measured on the basis of several indicators) and oxidative damage (as evidenced by significant decrease of MDA and hydrogen peroxide-the 2 key indicators of damage), along with significant upregulation of various enzymatic antioxidants. At the same time, osmolytes (GABA, proline and glycine betaine) showed significant enhancement too due to enhanced activity of their biosynthetic enzymes in heat primed and GABA treated plants. Photosynthetic activity, measured as chlorophyll concentration, PS II function, sucrose, and sucrose synthase enzyme, was noticeably higher in plants grown with primed seeds and GABA treatment. Consequently, the reproductive function (pollen, stigmatic and ovular activity) was significantly more in heat-stressed lentil plants, resulting in considerable improvement in yield traits, such as pod number per plant (21–24% and 35–38% in heat tolerant and heat sensitive genotypes, respectively) and seed yield per plant (22–24% and 37–40% in heat tolerant and heat sensitive genotypes, respectively). The present study clearly indicated that various biochemical traits—photosynthetic function, endogenous GABA, proline, glycine betaine and their biosynthetic enzymes, sucrose and its biosynthetic enzyme—were significantly upregulated with combined treatment of heat priming and GABA. There were some positive but weak associations with enhanced yield traits. There might be numerous other mechanisms affecting the yield traits under heat stress, which may need further investigation. These yield responses to treatments may look small, but considering the severe stress conditions and the fact that the plants were grown under controlled environments suggests opportunities. These findings need to be further validated under field conditions using realistic environments and under different stress levels with a greater number of genotypes. 

### 3.1. Impacts of Heat Stress

Heat stress impacted the lentil plants at various levels of organization, as noticed in the present study. Loss of membrane integrity because of heat stress can be attributable to disruption of lipid–protein interactions in membranes [24] and is similar to our previous studies on heat-stressed chickpea [25] and lentil [7] plants. The reductions in leaf water status possibly occurred because of decrease in stomatal conductance and hydraulic conductivity [26] and match the earlier observations in heat-stressed lentil [7]. Damage to mitochondrial integrity and denaturation or inhibition of respiratory enzymes [6] might have resulted in decrease in cellular oxidizing ability in heat-stressed lentil plants, which agrees with the observations on heat-stressed plants of cotton [27], wheat [28], chickpea [25] and potato (*Solanum tuberosum* L.) [29].

Heat stress resulted in significant reduction in chlorophyll (Chl) causing chlorosis at stage 2, more so in heat-sensitive genotypes, which was likely because of inhibition in chlorophyll biosynthesis or increase in its degradation of chlorophyll and/or disorganization of chloroplasts because of photooxidation [30]. Similar observations have been reported in plants of chickpea (*Cicer arietinum* L.) [25], tomato (*Solanum lycopersicum* L.) [31], and mungbean (*Vigna radiata* L.) [5] subjected to heat stress. Inhibition of PSII (photosystem II) function because of heat stress indicates disrupted electron transport and the denaturation of the oxygen-evolving enzymes of PSII light reactions of photosynthesis. PS II function has been used as a vital measure of thermotolerance [32]. Consequently, photosynthetic ability was inhibited, which was also associated with decrease in activity of sucrose-synthesizing enzyme (sucrose-P-synthase; SPS), resulting in impaired sucrose production in the present study, which agrees with previous findings in heat-stressed chickpea [33]. The disruption of pollen function (germination and viability), stigma and ovular activity, more so in heat-sensitive than heat-tolerant genotypes, confirmed our previous findings in heat-stressed lentil [7]. Diminished sucrose levels in the leaves of heat-stressed lentil plants and its transport might have inhibited the reproductive function [7] resulting in poor pod number. 

The osmolytes such as Pro, GB, and GABA showed significant increase in their concentration because of heat stress, pertinently in HT genotypes, while a marked reduction in their endogenous concentration was noticed in HS genotypes, as the stress progressed. Osmolytes tend to maintain homeostasis and perform diverse defense-related functions in the stressed cells [34,35,36], thus, poor levels of these osmolytes could be one of the reasons contributing to greater sensitivity of HS genotypes to heat stress. The reduction in Pro and GB concentration in HS genotypes correlated with inhibition of their biosynthesizing enzymes (P5CS and BADH, respectively). 

The oxidative molecules, such as Malondialdehyde (MDA) and hydrogen peroxide (H_2_O_2_), increased markedly in heat-stressed plants, and more in heat-sensitive genotypes. The increase in oxidative molecules might have caused membrane damage, chlorosis, necrosis, and loss of mitochondrial and chloroplast integrity in our study, which is similar to findings in heat-stressed wheat [37], chickpea [38], and lentil [39]. Cells control their redox status by up-regulating various enzymatic and non-enzymatic antioxidants [40]. Heat stress reduced the expression of these antioxidants, particularly at stage 2, more in heat-sensitive than heat-tolerant lentil genotypes, resulting in higher levels of oxidative molecules. Heat stress can impair the antioxidative mechanism by denaturing enzymes and reducing substrate availability [41], thus causing damage to various cellular components and tissues.

These adverse effects of heat stress resulted in reduction in yield traits (number of pods, seeds), which were more severe in heat-sensitive genotypes because of greater damage to reproductive and cellular function, as described above. 

### 3.2. Effect of Treatments

Due to heat stress, endogenous GABA concentration showed a marked reduction in HS genotypes as the stress progressed, while HT genotypes retained high GABA levels. GABA has been reported to improve leaf photosynthesis, stabilize membranes, and upregulate antioxidants and osmoprotectants in stressed cells [17,42]. Thus, diminution of endogenous GABA concentration in HS genotypes, under heat stress, might have been a critical factor increasing the damage to leaf tissues and reproductive function [43]. In the present study, we investigated the involvement of GABA in deciding the heat sensitivity. Hence, we explored whether heat priming the lentil seeds and foliar GABA application, alone or in combination, could increase the endogenous GABA concentration and thus improve the performance of heat-stressed lentil plants. Results indicated that HPr and GABA treatments considerably increased the endogenous GABA concentration, and effectively protected lentil plants from heat injury, especially when applied in combination (HPr+GABA). While the exogenous GABA treatment might have directly complemented its endogenous concentration, the increase because of heat priming might have occurred because of enhanced expression of enzyme glutamate decarboxylase, involved in GABA synthesis, as noticed in cold-acclimated wheat and barley (*Hordeum vulgare* L.) plants [44], which needs to be probed further. Here, we heat primed the hydrated seeds, instead of the plants (as done previously in some studies), to test the efficacy of this treatment on subsequent heat tolerance as well as to increase the practical utility of this technique, which proved to be effective in enhancing the heat stress tolerance in lentil plants. These findings are in consonance with some earlier studies reporting the beneficial effects of heat priming treatment (by subjecting the plants at the vegetative stage to moderately high temperature) on subsequent high temperature tolerance in *Arabidopsis* [13] and wheat [12]. Heat priming the seeds may induce ‘stress memory’ since the plants have evolved suitable mechanisms to remember the previous stress events and can react to subsequent stress events more quickly and strongly. These mechanisms include epigenetic changes, transcriptional priming, changes in proteins’ confirmation and metabolic and hormonal ‘signatures’ [45].

The combination of HPr and exogenous GABA was more effective in this regard and resulted in significantly improved leaf water status and stomatal conductance, particularly during stage 2. This might possibly be related to the facilitation of osmolytes’ accumulation, such as proline and GB, attributable to improved activity of their biosynthetic enzymes. Thus preventing damage to membranes and cellular oxidizing ability in the leaves. Osmolytes, besides contributing to turgor generation, have several other protective roles in stressed cells [46]. As a result, the leaves of plants treated with HPr+GABA combination showed less damage to chlorophyll thus preventing injury to photosynthetic activity (as PS II function). Mitigation of damage to leaves by this combined treatment might also be ascribed to reduced oxidative stress, associated with enhanced expression of antioxidants, as noticed in heat-stressed rice [17], salt-stressed maize (*Zea mays* L.) [19], and cold-stressed peach [47]. Our observations also match a previous study where heat-acclimated wheat plants showed less disruption in PSII function in a heat-stressed environment [48]. The HPr+GABA treatment contributed to stabilization of photosynthetic function causing optimization of sucrose production, as indicated by activity of sucrose synthesizing enzyme and sucrose concentration, which is considered critical to sustain vegetative and reproductive growth [33]. In a previous study in *Arabidopsis*, heat priming the plants was found to increase sucrose accumulation [13], which was associated with enhanced thermotolerance. Thus, the reproductive function in heat-stressed lentil plants was significantly improved by combined treatments, possibly because of enhanced sucrose availability to flowers and their components, which might have minimized the impact of heat stress to pollen function, resulting in an improved pod set [25]. The direct involvement of GABA in maintaining pollen fertility has been indicated earlier [23], which supports our observations. Thus, the present study showed that HPr and GABA treatments, especially when applied together, improved the pod number and seed yield of lentil plants under heat-stress.

## 4. Materials and Methods

### 4.1. Plant Growth Conditions, Treatments, Phenology and Yield Traits

#### 4.1.1. Plant Growth Conditions

**Figure 10:**
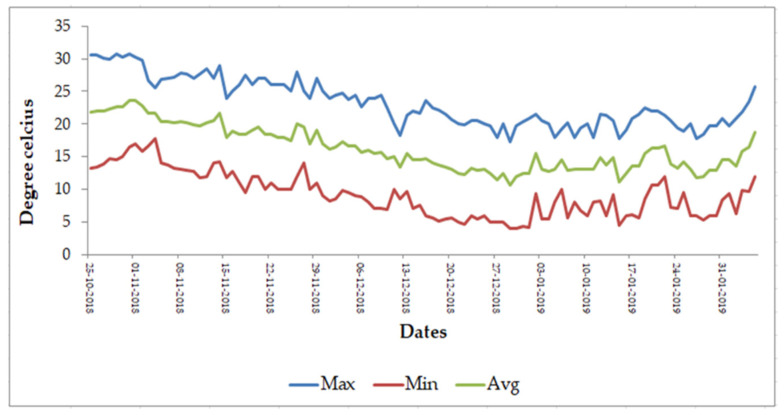
Temperature profile (maximum (Max), minimum (Min) and average (Avg)) from sowing to onset of flowering in outdoor environment at the experimental site.

Seeds of four lentil genotypes (two heat-tolerant (IG2507, IG3263), two heat-sensitive (IG2821, IG2849)), procured from the Indian Institute of Pulses Research, Kanpur, Uttar Pradesh, India; phenology (Figure 1), were heat primed at 35 °C for 6 h in the dark in Petri-dishes having double-layered filter paper moistened with distilled water (slow hydration) in a growth chamber maintained at 35 °C and relative humidity of 90% in the dark. A preliminary experiment tested the priming time (2, 4, 6, and 8 h) and temperatures (30, 32, and 35 °C), and determined 6 h at 35 °C to be appropriate for improving the performance of plants at 32/20 °C. For ‘control’ and ‘heat-stress alone’ treatments, the seeds were put in the dark in Petri-dishes with double-layered filter papers, moistened with distilled water for 6 h in a controlled environment at 28/18 °C (day/night). The seeds were sown directly after priming, as a preliminary study revealed that drying the seeds to original moisture reduced the effectiveness of the treatment. The primed seeds were sown in pots (10 cm diameter; 7 kg capacity) filled with a sandy loam soil (sand: silt: clay in 63.4%, 24.6% and 12%) mixed with sand in a 3:1 ratio. *Rhizobium spp.*, specific to lentil, was added to the soil prior to sowing. Soil used in the present study had a mixture (3:1) of soil–sand and part farmyard manure, 10 mg kg^−1^ of tricalcium phosphate [38]. The plants were raised in October at Panjab University, Chandigarh, India in a natural environment (see Figure 10 for temperature data) until the onset of flowering (105–107 days after sowing depending on the genotype). The average temperature was 25/15 °C (day/night), light intensity ranged from 1350–1550 µmol m^–2^ s^−1^ and mean relative humidity (RH) values ranged from 62–69%.

#### 4.1.2. Treatments

At the onset of flowering, one set (half) of plants was maintained in a controlled environment at 28/18 °C (day/night, 12 h each), light intensity of 500 µmol m^−2^ s^−1^, and RH of 65–70%, while the other set was subjected to heat stress (32/20 °C, day/night; 12 h each) up to maturity, with similar light and RH values to the control. For the heat-stress treatment, the plants were kept initially at 28/18 °C for one day, before gradually increasing the temperature by 2 °C/day to achieve the desired temperature (32/20 °C; day/night; 12 h each). The plants were treated with a foliar application of GABA (1 mM), along with Tween 20 (as a surfactant), one day before final exposure to heat stress (32/20 °C; day/night; 12 h each), and again five days later. 

The treatments were as follows:

1. Control;
2. Heat-stress alone;
3. Heat-primed seeds + Heat-stress;
4. Heat-stress + GABA (1 mM) as a foliar treatment;
5. Heat-primed seeds + Heat-stress + GABA (1 mM) as a foliar treatment.

The plants were assessed for various leaf traits at stage 1 (6th day of exposure to 32/20 °C) and stage 2 (15th day of exposure to 32/20 °C). Phenology was recorded during different growth stages, while yield traits were examined at maturity.

#### 4.1.3. Phenology and Yield Traits

Phenology observations (number of days taken to show flowering, podding, maturity, flowering–podding interval, podding–maturity interval; Figure 1) were recorded on five plants per genotype in each replicate (15 plants per genotype), pooled and averaged. Mature seeds were harvested for recording yield data, the seeds were dried at 45 °C in hot air oven for three days; these were weighed, and the average values were recorded on the basis of per plant [33].

### 4.2. Stress Injury

#### 4.2.1. Membrane Damage (as Electrolyte Leakage)

Fresh leaves (100 mg) (young, 2–3rd node from the top; for all the traits examined) beneath to the flowers were collected at both stages to measure electrolyte leakage [49]. Leaf segments were cleaned with water (deionized), kept in glass vials (capped) with ten mL deionized water at 25 °C for 12 h. The electrical conductivity (C1) of the surrounding solution was recorded after 24 h. Subsequently, leaf segments were placed for 10–15 min in a water bath (maintained at 80 °C). The final reading of electrical conductivity reading (C2) was recorded upon equilibration [25]. The values for electrolyte leakage were measured using the equation C1/C2 × 100 and expressed as percentage.

#### 4.2.2. Relative Leaf Water Content

Relative leaf water content (RLWC) was measured to assess the leaf water status [50]. Leaves subtending flowers (100 mg; fresh weight (FW)) were collected, floated in a Petri dish having distilled water for 2 h, thereafter, these were taken out, and dried of their surface with filter paper; these were weighed again (turgid weight, TW) before oven-drying for 24 h at 110 °C; these were weighed again for dry weight (DW). It was calculated as (FW−DW)/(TW−DW) × 100; expressed as percentage.

#### 4.2.3. Stomatal Conductance

Stomatal conductance (gs) of leaves beneath the flowers was measured with a portable leaf porometer (Decagon Devices, Pullman, Washington, USA) was used to measure [25], and expressed as mmol^−1^ s^−1^.

#### 4.2.4. Cellular Oxidizing Ability

Cellular oxidizing ability was assessed using TTC (2, 3, 5-triphenyl tetrazolium chloride) reduction ability. Fresh leaf samples (100 mg) were excised into small segments and dipped in incubation solution containing sodium phosphate (pH 7.4; 50 mM), TTC (500 mg 100 mL^−1^ solution) [51]. The leaf samples were placed in dark at 25 °C for one hour without shaking as TTC reduction is responsive to excessive oxygen. After extracting twice with 5 mL of 95% ethanol, the extracts were pooled to make final volume of 10 mL. The color developed due to production of Formazan was read with a Spectrophotometer at 530 nm, rather than 485 nm, to minimize any interference by pigments like chlorophyll [25]. The readings are given as absorbance/g fresh weight.

### 4.3. Reproductive Function

#### 4.3.1. Pollen Germination

The germination of pollen grains was tested in a growth medium with potassium nitrate (990 mM; pH 6.5), calcium nitrate (1269 mM), magnesium sulfate (812 mM), sucrose (10%), and boric acid (1640 mM) [25,52]. Pollen grains were recorded as germinated when the pollen tube size increased more than the pollen grain’s diameter. The germination was measured from about hundred pollen grains per replicate. 

#### 4.3.2. Pollen Viability

Pollen viability was tested using 0.5% acetocarmine, involving about 200 pollen grains in 5 microscopic fields [25]. The collection of the pollen grains was done from flowers on day of anthesis, and the pollen grains were combined and examined for their viability [53]. The traits used for measuring pollen viability were size and shape (triangular or spherical) and the color intensity of the pollen grains. Dense color indicated higher pollen viability [25], expressed as percentage.

#### 4.3.3. Stigma Receptivity

Stigma receptivity was examined by esterase test following the method of [54]. A day prior to opening of flower, stigmas were harvested from the flowers. These were placed at 37 °C for 15 min in a solution with α-NAA and fast blue B prepared in phosphate buffer. The stigmas develop colors of varying intensity depending upon their receptivity and are rated on 1–5 scale (5-high receptivity, 1-low receptivity) [25].

#### 4.3.4. Ovule Viability

For testing ovule viability, the ovules were harvested from the ovary of flowers one day before anthesis, which were kept on a slide containing few drops of TTC solution (0.5% TTC in 1% sucrose solution), covered with a cover slip, and placed in a Petri-dish containing double layered moistened filter paper. These were incubated at 25 °C in the dark for 15 min in a chamber. The ovules were tested for viability on the basis of red color intensity of the stain, particularly in the center. The color intensity depends upon the respiring ability of the ovules and is rated on 1–5 scale (5-highest intensity, 1-lowest intensity) [25].

### 4.4. Leaf Photosynthetic Function

#### 4.4.1. Photochemical Efficiency 

For this purpose, chlorophyll fluorescence (as Fv/Fm ratio) was measured from the young leaves, close to flowers, using chlorophyll fluorometer OS1-FL (Opti-Sciences, Hudson, NH, USA) [25].

#### 4.4.2. Chlorophyll

For extracting chlorophyll, fresh leaves (500 mg) were extracted using 80% acetone, and the extract was centrifuged at 5702× *g*. The supernatant was collected, and its absorbance was read in a spectrophotometer at 645 and 663 nm [55], expressed as mg g^−1^ dry weight.

#### 4.4.3. Sucrose

For measuring sucrose concentration, extraction of fresh leaves (500 mg) was done in 80% ethanol for 1.5 h at 80 °C two times, followed by pooling of these extracts. These were evaporated in oven (air-circulating) at 40 °C and tested for sucrose concentration [56], as detailed previously [25], expressed as µmoles g^−1^ dry weight.

#### 4.4.4. Sucrose Phosphate Synthase

Fresh leaves (beneath the flowers) were collected and extracted in a chilled 50 mM HEPES buffer-NaOH (pH 7) with MgCl_2_ (2 mM), EDTA (1 mM), and DTT (2 mM) [57]. Sephadex G-25 columns, kept at 4 °C, were used for desalting of the supernatant. Prior to this, these columns were pre-equilibrated using a buffer having HEPES-NaOH (pH 7.5; 20 mM), MgCl_2_ (0.25 mM), 2-mercaptoethanol (0.01%), ethylenediaminetetraacetic acid (EDTA; 1 mM), and BSA (0.05%). To assay the enzyme activity, from this extract, anthrone test was used [58], as detailed previously [7]. The activity was expressed as µmoles sucrose produced g^−1^ dry weight h^−1^.

### 4.5. Soluble Proteins

Oven-dried leaves were homogenized in 0.1 M phosphate buffer (pH 7.0), followed by centrifugation at 514 g for 15 min [7]. The concentration of soluble proteins was quantified following the method of [59]. These were expressed as mg g^−1^ dry weight.

### 4.6. Osmolytes and Related Enzymes

#### 4.6.1. Proline and Pyrroline-5-Carboxylate Synthase

For measuring proline, the leaf tissue was extracted using 3% sulphosalicylic acid, and it was centrifuged 20 min at 4 °C at 2150 g. The supernatant was treated with acidic ninhydrin reagent and read at 520 nm; toluene was used a blank [60]. The concentration was expressed as nmoles g^−1^ dry weight.

To measure pyrroline-5-carboxylate synthase (P5CS) activity, tissue samples were homogenized in potassium phosphate buffer (0.1 M; pH 7.5) with mercaptoethanol (10 mM), EDTA (1 mM), polyvinylpyrrolidone (1% (m/v), KCl (0.6 M), MgCl_2_ (5 mM) in a pre-cooled pestle and mortar. The extract was centrifuged at 3360 g for 30 min at 4 °C in a cold centrifuge. The desalting of the enzyme extract was done in Sepahdex columns at 4 °C; the enzyme activity was measured [61] and expressed as nmoles NADP formed min^−1^ mg^−1^ protein.

#### 4.6.2. Glycine Betaine and Betaine Aldehyde Dehydrogenase

Leaf tissue was dried in an oven and crushed to make fine powder, before adding twenty ml of deionized water, and shaking at 25 °C for 24 h. Using 2 N H_2_SO_4_, the extracts were diluted (1:1) and measured for glycine betaine concentration [62]. The concentration was expressed as µmoles g^−1^ dry weight.

Briefly, the activity of the enzyme betaine aldehyde dehydrogenase (BADH) was assayed as follows. The leaf tissue was extracted in a medium with HEPES-KOH buffer (pH 8.0; 50 mM), EDTA (1 mM), dithiothreitol (5 mM), ascorbic acid (5 mM), sodium borate (10 mM), sodium metabisulfite (20 mM), and PVP [2% (*w*/*v*)]. The extract was centrifuged at 4 °C for 15 min at 3360× *g*. After desalting the enzyme extract, the activity was measured at 340 nm [63]. The activity was expressed as U mg^−1^ protein,

#### 4.6.3. Endogenous GABA

Leaf tissue was extracted in 8% (*m*/*v*) trichloroacetic acid (TCA) at 3360× *g* for 1 min at 25 °C, and then centrifuged for 20 min at 3360× *g*. The supernatant was collected, followed by addition of 4 mL pure diethyl ether, mixing briskly for 10 min, and centrifuging for 20 min at 3360× *g*. The supernatant after collection was allowed to stand in the open air for evaporation of ether for 30 min and tested for GABA concentration [64], which was expressed as µmoles g^−1^ dry weight.

### 4.7. Oxidative Molecules and Antioxidants

#### 4.7.1. Malondialdehyde

For, malondialdehyde (MDA), fresh leaf tissue was extracted in trichloroacetic acid (TCA; 0.1%), followed by centrifugation for 5 min at 3360× *g*. The reaction was carried out by mixing 0.1 mL of supernatant with thiobarbituric acid (4 mL; 0.5%), prepared in TCA (20%). The contents were heated for 0.5 h at 95 °C, which were subsequently cooled in an ice bath. It was centrifuged at 3360× *g* for 10 min at 4 °C, followed by recording its absorbance at 532 nm. The extinction coefficient of 155 mM^−1^ cm^−1^ was used for calculating the concentration of MDA [65], which was expressed as nmoles g^−1^ dry weight.

#### 4.7.2. Hydrogen Peroxide

Fresh plant tissue was extracted in 5 mL chilled acetone (80%), the extract was filtered using a Whatman filter paper. The filtrate was reacted with titanium reagent (4 mL), followed by addition of ammonia solution (25%, 5 mL). It was centrifuged at 3360 *g*; the supernatant was discarded, followed by dissolution of the residue 1 M H_2_SO_4_. The optical density of the resultant solution was read at 410 nm. The H_2_O_2_ concentration was measured with the help of the extinction coefficient of H_2_O_2_ (0.28 mmol^−1^ cm^−1^) [66] and expressed as expressed as nmoles g^−1^ dry weight.

#### 4.7.3. Superoxide Dismutase

For assaying superoxide dismutase (SOD; E.C. 1.15.1.1) activity from fresh leaf tissue, the extraction was done in a pre-cooled phosphate buffer (50 mM; pH 7.0), followed by centrifugation (3360 g) 4 °C for 5 min. The supernatant was tested for enzyme activity. The reaction mixture comprised of phosphate buffer (pH 7.8; 50 mM), enzyme extract (0.1 mL), sodium bicarbonate (50 mM), nitro blue tetrazolium chloride (NBT; 25 mM), methionine (13 mM), EDTA (0.1 mM) in an entire volume of three mL. Subsequently, after addition of riboflavin (2 mM), the reaction mixture was exposed for 10 min to a 15 W fluorescent light. The absorbance was taken at 560 nm. The enzyme activity was measured using the method of Dhindsa and Matowe [67], which was expressed as Units mg^−1^ protein.

#### 4.7.4. Catalase

The activity of Catalase (CAT; E.C. 1.11.1.6) was assayed as per Teranishi et al. [68]. The enzyme extract prepared for assaying the SOD activity was also used for CAT activity. To the reaction mixture ((enzyme extract (0.1 mL) and phosphate buffer (pH 7.0; 50 mM)), H_2_O_2_ (200 mM) was included to start the reaction. The optical density (at 410 nm) was read for 3 min. The activity of enzyme was determined by means of extinction coefficient of 40 mM^−1^ cm^−1^ and expressed as mmol H_2_O_2_ decomposed mg^−1^ protein.

#### 4.7.5. Ascorbate Peroxidase

The reaction mixture (3 mL) comprised of enzyme extract, which was prepared for SOD, phosphate buffer (pH 7.0; 50 mM), EDTA (0.1 mM) and ascorbic acid (ASC; 0.5 mM). Subsequently, hydrogen peroxide (as a substrate) was added to the reaction mixture. Ascorbate peroxidase (APX; E.C. 1.11.1.11) activity was measured as the decline in absorbance at 290 nm by recording the oxidation of ascrobate and calculated using the extinction coefficient of 2.8 mM^−1^ cm^−1^ [69]. The activity was expressed as mmol oxidized donor decomposed min^−1^ mg^−1^ protein.

#### 4.7.6. Glutathione Reductase

For assaying glutathione reductase (GR; E.C. 1.6.4.2), the reaction mixture had phosphate buffer (1.5 mL; 100 mM, pH 7.6), BSA (0.20 mL), NADP (0.35 mL), glutathione oxidized (GSSG; 0.1 mL), and enzyme extract (0.1 mL; as above for SOD). The activity was assayed as decline in absorbance for 3 min at 340 nm [70] and expressed as mmol oxidized donor decomposed min^−1^ mg^−1^ protein.

### 4.8. Statistical Analysis

The experiment had four contrasting genotypes (two heat-tolerant and two heat-sensitive) and four treatments. Each treatment was comprised of eight pots per genotype (two plants per pot) in triplicate (24 pots per treatment; 48 plants per treatment). Three pots in triplicate (nine plants per treatment; 18 plants per genotype) were maintained separately for yield trait measurements. The pots were kept following a randomized block design in controlled environment. All the traits were analyzed in 3 replicates. Analysis of variance (ANOVA) for genotypes × treatment × stages interaction was conducted using Agristat software, and the least significant values (LSD) values were calculated (*p* < 0.05). Tukey’s post hoc test was used to compare means.

## 5. Conclusions

The present study revealed that HPr and exogenous application of GABA increased the endogenous GABA concentration, along with several other defense-related mechanisms to reduce the heat stress injury to the leaves and reproductive function resulting in improved yield-related traits. Thus, maintaining an appropriate endogenous GABA concentration might be a vital mechanism associated with heat tolerance in lentil at reproductive stages. The findings showed for the first time that combining heat priming of the hydrated seeds with foliar GABA treatment provides an opportunity to reducing the effects of heat stress in lentil. Further research is needed to validate these studies under field conditions, find a more practical and simple method of heat priming, determine the economics of cost and return on investment associated with heat priming and GABA application under field conditions.

## Data Availability

Data available upon request from corresponding author.
